# Identification and cross-validation of genetic loci conferring resistance to Septoria nodorum blotch using a German multi-founder winter wheat population

**DOI:** 10.1007/s00122-020-03686-x

**Published:** 2020-10-12

**Authors:** Min Lin, Melanie Stadlmeier, Volker Mohler, Kar-Chun Tan, Andrea Ficke, James Cockram, Morten Lillemo

**Affiliations:** 1 Department of Plant Sciences, Norwegian University of Life Sciences, Post Box 5003, 1432 Ås, Norway; 2 Bavarian State Research Center for Agriculture, Institute for Crop Science and Plant Breeding, Freising, Germany; 3 Centre for Crop and Disease Management, School of Molecular and Life Sciences, Curtin University, Bentley, WA Australia; 4 Norwegian Institute of Bioeconomy Research, Høgskoleveien 7, 1433 Ås, Norway; 5 John Bingham Laboratory, NIAB, 93 Lawrence Weaver Road, Cambridge, CB3 0LE UK

## Abstract

**Key message:**

We identified allelic variation at two major loci, *QSnb.nmbu-2A.1* and *QSnb.nmbu-5A.1*, showing consistent and additive effects on SNB field resistance. Validation of *QSnb.nmbu-2A.1* across genetic backgrounds further highlights its usefulness for marker-assisted selection.

**Abstract:**

Septoria nodorum blotch (SNB) is a disease of wheat (*Triticum aestivum* and *T. durum*) caused by the necrotrophic fungal pathogen *Parastagonospora nodorum*. SNB resistance is a typical quantitative trait, controlled by multiple quantitative trait loci (QTL) of minor effect. To achieve increased plant resistance, selection for resistance alleles and/or selection against susceptibility alleles must be undertaken. Here, we performed genetic analysis of SNB resistance using an eight-founder German Multiparent Advanced Generation Inter-Cross (MAGIC) population, termed BMWpop. Field trials and greenhouse testing were conducted over three seasons in Norway, with genetic analysis identifying ten SNB resistance QTL. Of these, two QTL were identified over two seasons: *QSnb.nmbu-2A.1* on chromosome 2A and *QSnb.nmbu-5A.1* on chromosome 5A. The chromosome 2A BMWpop QTL co-located with a robust SNB resistance QTL recently identified in an independent eight-founder MAGIC population constructed using varieties released in the United Kingdom (UK). The validation of this SNB resistance QTL in two independent multi-founder mapping populations, regardless of the differences in genetic background and agricultural environment, highlights the value of this locus in SNB resistance breeding. The second robust QTL identified in the BMWpop, *QSnb.nmbu-5A.1,* was not identified in the UK MAGIC population. Combining resistance alleles at both loci resulted in additive effects on SNB resistance. Therefore, using marker assisted selection to combine resistance alleles is a promising strategy for improving SNB resistance in wheat breeding. Indeed, the multi-locus haplotypes determined in this study provide markers for efficient tracking of these beneficial alleles in future wheat genetics and breeding activities.

**Electronic supplementary material:**

The online version of this article (10.1007/s00122-020-03686-x) contains supplementary material, which is available to authorized users.

## Introduction

Wheat is one of the most important staple food sources worldwide, with gross production valued at around 168 billion US dollars (Food and Agriculture Organization of the United Nations 2016). However, wheat production is threatened by various bacterial, fungal and viral diseases. *Parastagonospora nodorum* is a devastating fungal pathogen of both bread wheat (*Triticum aestivum*) and durum wheat (*T. durum*) with disease epidemics reported in nearly all wheat producing regions with warm and humid growing conditions (Oliver et al. 2012; Francki 2013; Ficke et al. 2018). By causing damage to both wheat leaves and ears, *P. nodorum* can reduce yield by up to 30% (Bhathal et al. 2003). So far, regardless of resistance breeding efforts, no cultivar has shown complete resistance to *P. nodorum* in the field, and control of SNB still largely depends on fungicide application (Duba et al. 2018). However, intensive use of fungicides increases the risk of fungicide resistance, and the resulting reduction in available modes of action challenges the effectiveness of future chemical control (Holloman 2015). Therefore, research on host genetic resistance is needed in parallel with efforts to find new modes of action for chemical control.

*P. nodorum* is the model organism for necrotrophic fungal pathogens and much research has been done to characterize the interactions between *P. nodorum* and wheat (Oliver et al. 2012). *P. nodorum* can trigger plant cell death and expand infections by secreting proteinaceous necrotrophic effectors (NEs) that target dominant susceptibility loci in the wheat host (Friesen et al. 2007). This interaction between pathogen effector and host sensitivity locus is termed the ‘inverse gene-for-gene model’ (Friesen et al. 2007). Accumulation of multiple susceptibility loci in a wheat cultivar may result in higher disease severity to certain *P. nodorum* isolates, as some wheat susceptibilities to NEs have been shown to be quantitative and additive (Friesen et al. 2009). To date, eight *P. nodorum* NEs have been characterized, which interact with nine susceptibility loci distributed over seven wheat chromosomes (Ruud and Lillemo 2018). Among those, three *P. nodorum* NE-coding genes (*ToxA, Tox1* and *Tox3*) (Liu et al. 2006, 2009, 2012) and two wheat susceptibility genes (*Tsn1* and *Snn1*) (Faris et al. 2010; Shi et al. 2016) have been cloned. When used in conjunction with traditional marker-assisted selection approaches targeting SNB resistance QTL, eliminating susceptibility alleles from wheat cultivars could be a potential strategy to enhance SNB resistance breeding. For example, in Australia the ToxA-*Tsn1* interaction was found to be a significant factor in field SNB susceptibility. Subsequent reduction of the ToxA sensitive wheat growing area by 13.5% between 2009 and 2013 was estimated to have saved 50 million $ in crop losses (Vleeshouwers and Oliver 2014). Genetic studies have identified numerous additional SNB resistance QTLs at the juvenile or the adult plant stages (reviewed most recently by Ruud and Lillemo 2018, with additional QTL identified in subsequent studies by Czembor et al. 2019; Francki et al. 2020; Hu et al. 2019; Lin et al. 2020a; Ruud et al. 2019), including many that have not been associated with genetic loci controlling effector sensitivity. Taken together, these map to 20 of the 21 wheat chromosomes. Therefore, phenotypic resistance in wheat is likely a result of selection against effector sensitivity alleles combined with selection for SNB resistance alleles.

Genotype by environment interaction commonly plays an important role in determining SNB resistance/susceptibility field phenotype. In addition, QTL identified using one mapping population may not necessarily be identified in another mapping population, due to differences caused by the genetic background (Langridge et al. 2001). Of the eight known NE susceptibility loci, four have been reported to co-locate with field SNB QTL: *Tsn1*, *Snn1*, *Snn2* and *Snn3-B1* (Friesen et al. 2009; Phan et al. 2016; Ruud et al. 2017; Ruud and Lillemo 2018). Compared to biparental populations, the genetic analysis of target traits using multi-parent advanced generation inter-cross (MAGIC) populations could be considered as more relevant for the characterization of QTL for use in breeding programs, as the multiple founders used (typically between 4 and 16) provide the possibility of capturing increased numbers of alleles at any given locus (Wei and Xu 2016; Scott et al. 2020), as well as efficiently combining founder haplotypes via multiple rounds of intercrossing. These properties of MAGIC populations allow resulting QTL to be assessed under a wider range of genetic backgrounds, and increases the chances of detecting disease resistance QTL within the framework of a single genetic mapping population (Cockram and Mackay 2018). Recently, MAGIC populations have begun to be used for numerous genetic studies of wheat disease resistance and fungal effector sensitivity (Cockram et al. 2015; Downie et al. 2018; Stadlmeier et al. 2019; Lin et al. 2020a; Corsi et al. 2020). A recent study by Lin et al. (2020a) investigated *P. nodorum* resistance at both the seedling and adult plant stages using a UK-relevant eight-founder wheat MAGIC resource, termed the ‘NIAB Elite MAGIC’ population (Mackay et al. 2014). Numerous QTL were identified, including *QSnb.niab-2A.3*, which was detected consistently across years and locations. Additionally, the observation that this QTL was identified using infiltration at the seedling stage with *P. nodorum* culture filtrate indicates that the adult plant SNB resistance conferred by this locus could be due to the mutation of an effector sensitivity allele (Lin et al. 2020a). The stability of *QSnb.niab-2A.3* indicated that resistance alleles at this locus could be a useful target for marker assisted selection (MAS) in SNB resistance breeding.

The ‘Bavarian MAGIC winter wheat population’ (BMWpop) is an eight-founder wheat MAGIC population of German origin. Evaluating SNB disease severity in the BMWpop, which has a partially different genetic background compared to the UK ‘NIAB Elite MAGIC’, may provide additional SNB resistance loci for germplasm enhancement. In addition, if common QTL could be detected between the two MAGIC populations, MAS for such QTL could be applied with increased confidence in wider European wheat breeding programs. Lin et al. (2020a) reported that ToxA-*Tsn1* and Tox3-*Snn3-B1* interactions showed effects on seedling *P. nodorum* resistance but were not represented among QTL detected at the adult plant stage by field testing. However, Lin et al. (2020a) found that using infiltration of culture filtrate (CF) from *P. nodorum* isolate 203649, which possessed uncharacterized effectors, detected a QTL that was also identified for adult plant resistance in the field across years and location, *QSnb.niab-2A.3* (Lin et al. 2020a). In summary, it appears that field SNB resistance is composed of interactions between known NEs and susceptibility loci, as well as additional QTLs for which the underlying mechanism controlling resistance remains unknown. The objectives of this study were to (1) identify SNB QTL in the German BMWpop MAGIC population by both seedling infiltration and field testing and compare these with QTL identified in the UK ‘NIAB Elite MAGIC’ population, (2) where phenotypic differences between the BMWpop founders for sensitivity to known *P. nodorum* effectors or CF are identified, phenotypically screen the population to investigate whether sensitivity QTL co-locate with adult plant SNB QTL, and (3) identify haplotypes and determine additive effects at the prioritized QTL that might help future breeding efforts to combine multiple sources of SNB resistance.

## Materials and methods

### Germplasm and genotypic data

The BMWpop and associated genotypic data have previously been described by Stadlmeier et al. (2018). Briefly, the population was developed at the Bavarian State Research Center for Agriculture (LfL) using eight founders (the German varieties Event, Format, BAYP4535, Potenzial, Bussard, Firl3565 and Julius, and Danish variety Ambition), selected based on multiple agronomic and disease resistance traits. The population consists of 394 F_6:8_ recombinant inbred lines (RILs). Together with the eight founders, the RILs were genotyped using a 15 K + 5 K Infinium iSelect single nucleotide polymorphism (SNP) array, which combined markers from the Illumina 90 K wheat SNP chip (Wang et al. 2014) and the 820 K Axiom array (Winfield et al. 2016). The resulting genotypic datasets were used by Stadlmeier and Hartl (2018) to make the BMWpop genetic map consisting of 5435 SNPs. These BMWpop resources were used here for QTL analysis of SNB resistance/sensitivity.

### Field trials

Hillplot (small plots sown 50 cm apart in rows, 40 cm between rows) trials were conducted for SNB leaf blotch over three seasons (2016, 2017, 2018) at Vollebekk research station in Ås, Norway. The germplasm used for each trial consisted of the BMWpop (394 RILs and eight founders) as well as three control varieties: Jenga (relatively high resistance), Arina (moderately resistant) and Tarso (susceptible). An augmented trial design was used where the eight founders and the three controls were laid out using an alpha lattice with eight replicates, giving two checks in each row of twelve plots. Then the RILs, mostly unreplicated, were distributed randomly in the remaining field plots. Field trials were sown in autumn, established over the winter, and phenotyped the following summer as they progressed to maturity. Naturally *P. nodorum*-infected straw harvested from the most susceptible lines in the previous field season were used as inoculum and was applied to the field trials before stem elongation in spring. Mist irrigation was applied for 5 min every half hour from 10 am to 8 pm to enhance infection. Mist irrigation started at the same time as the inoculum was applied to the field and ended after the final scoring had been done. The selective fungicide Forbel 750 (Bayer Crop Science, a.i.: phenpropimorph) was applied to the field trials every three weeks at the full recommended dose rate to control infections of stripe rust and powdery mildew. Forbel 750 has little to no effect on *P. nodorum* infection.

### Phenotypic evaluation of SNB leaf blotch severity in the field

SNB leaf blotch severity was scored via visual estimation of the percentage of diseased leaf area in each hillplot canopy. In 2016 and 2017, leaf blotch severity was assessed three times. The first disease scoring was carried out when the diseased area of the canopy reached around 50% for the most susceptible lines/controls, followed by approximately weekly assessments. Due to hot and dry weather, plant development was strongly accelerated in 2018, resulting in disease scoring being undertaken only twice. The first scoring followed the same criteria as described above, while the second scoring was done when the most susceptible lines reached 100% disease severity. Plant height (PH) and days to heading (DH) were also assessed each year. PH (cm) was measured from ground to bottom of the wheat ears, and DH was scored when most plants within a hillplot had ears fully emerged (Zadoks` growth stage 55, GS55).

### Culture filtrate and Tox3 preparation

The *P. nodorum* isolate 203649 used for culture filtrate infiltration of the BMWpop was the same as described by Lin et al. (2020a). This isolate was selected to screen the BMWpop as it is a local Norwegian isolate relevant to our field trial locations in Norway. Furthermore, this isolate does not produce any of the three cloned effectors (ToxA, Tox1 and Tox3), thus potentially helping the identification of novel effector sensitivity loci beyond *Tsn1, Snn1* and *Snn3-B1*. Following the methods described by Friesen and Faris (2012), the isolate was grown in liquid Fries 3 medium and after three-week stationary growth, the culture filtrates were filter-sterilized. Previous studies showed that sensitivities to ToxA and Tox3 were positively correlated with SNB susceptibility in the field in Norway (Ruud et al. 2017, 2018). However, all eight founders of BMWpop are insensitive to the ToxA effector. Therefore, infiltration experiments for ToxA were not carried out in this study. For Tox3 effector production, Tox3 was expressed in *Pichia pastoris* as described by Tan et al. (2014). The semi-purified Tox3 effector was desalted in 20 mM sodium phosphate (pH 7.0) and freeze-dried for storage. Prior to use, ultra-pure water was used for re-suspension of Tox3.

### Seedling infiltration

Three to four seeds of each of the BMWpop RILs and the eight founders were sown in cones (Stuewe and sons, Tangent, Orlando, USA) filled with peat soil (Gartnerjord, Tjerbo, Norway). Seedlings were grown in a greenhouse with 16 h light per day, temperature 20/16 °C (day/night) and 65% relative humidity for 14 days. Approximately 50 μL of the culture filtrate or Tox3 effector was infiltrated into the second leaf of two-week-old seedlings using a 1-mL syringe with needle removed. Reactions to culture filtrate or Tox3 effector were scored seven days after infiltration using a 0–3 scale (Friesen and Faris 2012) where 0 represents completely insensitive, 1 represents mottled chlorosis, 2 represents complete chlorosis, and 3 represents necrosis. The experiment was conducted with three biological replicates of each RIL and six to nine biological replicates for each of the eight founders.

### Statistical analysis

The PROC MIXED procedure in SAS v.9.4 (SAS Institute Inc.) was used to calculate mean disease severity, PH and DH of each genotype within each year. For the analysis of the field trials within each year, multi-linear regression with PH and DH as covariates was carried out in RStudio version 1.1.442 (RStudio Team 2015) to determine whether PH and/or DH affected leaf blotch disease severity with the model:

$$ {\text{Mean disease severity }} = {\text{ DH }} + \, \left( {{\text{PH}}} \right) \, + \, \left( {{\text{DH }} \times {\text{ PH}}} \right) $$

The corrected disease severities were obtained by subtracting the residuals from the linear regression model with PH and/or DH as covariate, when PH and/or DH was significantly (*p* < 0.0001) correlated with leaf blotch disease severity using “resid” function by R. Shapiro–Wilk tests were carried out in RStudio to test for normality of mean/corrected disease severity.

### QTL analysis

A subset of 2804 SNP markers previously assigned to unique map positions in the BMWpop genetic map (Stadlmeier et al. 2019) were used for interval mapping (IM) and composite interval mapping (CIM). Founder probabilities were calculated using the function ‘mpprob’ in the R/mpMap package V2.0.2 (Huang and George 2011) at the threshold of 0.7. IM was carried out using the function ‘mpIM’ in R/mpMap with the founder haplotype probabilities obtained from the previous step. CIM was undertaken using either 5 (CIM-cov5) or 10 (CIM-cov10) cofactors. 1000 simulations of the phenotypic dataset were conducted and used to obtain an empirical distribution of genome-wide significance p values based on a null QTL hypothesis. The significance threshold was then determined by the genome-wide significance p value at the threshold level α < 0.05, on a trait by trait basis. All detected QTL were then fitted in a full model using the function ‘fit’ to obtain additive founder effects (relative to the founder, Julius) and the phenotypic variation (*R*^2^) explained by each QTL. The supporting interval of each QTL was defined as markers with −log_10_(*p*) value ± 0.5 of the peak marker’s −log10(*p*) value. CIM-cov5 and CIM-cov10 were carried out to further confirm and refine the genetic map locations of the QTL detected by IM. In addition, QTL mapping via identity-by-descent (IBD) analysis was undertaken to support the outcome of IM using all 5435 mapped SNP markers, based on a regression model against the founder haplotype probabilities of each marker. The founder haplotype probabilities were calculated as described above, and the additive founder effects were estimated relative to the founder, Julius. R/q value package was used to correct for multiple testing of IBD with a significant threshold *q* = 0.05. Flanking DNA sequences for SNP markers were obtained from websites https://triticeaetoolbox.org and https://www.cerealsdb.uk.net. Physical map positions of SNP markers in the cv. Chinese Spring wheat reference genome assembly, RefSeq v1.0 (International Wheat Genome Sequencing Consortium (IWGSC) et al. 2018), were obtained via BLASTn analysis using the website https://urgi.versailles.inra.fr/blast_iwgsc/?dbgroup=wheat_iwgsc_refseq_v1_chromosomes&program=blastn. Genetic linkage groups overlaid with the positions of QTL intervals were graphically displayed using Mapchart 2.32 (Voorrips 2002).

### Haplotype analysis for QTL *QSnb.nmbu-2A.1* and *QSnb.nmbu-5A.1*

Five markers within the *QSnb.nmbu-2A.1* QTL interval from 2018 (*QSnb.nmbu-2A.1/2018*) and with the highest −log_10_(*p*) values were selected to construct haplotypes. Six most significant markers located at the overlapping supporting interval of *QSnb.nmbu-5A.1* detected in both 2016 and 2018 were selected to construct haplotypes. As the haplotype effect of *QSnb.nmbu-5A.1* was contributed mainly by allelic differences at marker *wsnp_Ex_c898_1738424*, for this QTL the comparison was based on the allele effect of marker *wsnp_Ex_c898_1738424* alone*.* The mean disease severities from 2016 and corrected disease severities from 2017 and 2018 for each haplotype in the BMWpop RILs were calculated and compared by Kruskalmc test (*p* < 0.05) using the R/pgirmess package (Giraudoux 2018). An additional haplotype analysis of QTL *QSnb.nmbu-2A.1/2016* was conducted using the peak marker *BobWhite_c3833_815* and the closely linked marker *AX-94825088* for validating the founder effect of the founder, Event.

### Analysis of additive effects

Genotypes possessing either only the susceptible haplotype 4 at *QSnb.nmbu-2A.1/2018* or only the susceptible allele for marker *wsnp_Ex_c898_1738424* at *QSnb.nmbu-5A.1,* were grouped together as carrying one resistant allele. Genotypes that carried both susceptible alleles were grouped as carrying zero resistant alleles, while the remaining genotypes were grouped as carrying two resistant alleles. Comparison of disease severities between genotype groups was conducted using the same method as described for haplotype analysis.

## Results

### Phenotypic evaluation of SNB field resistance

**Fig. 1:**
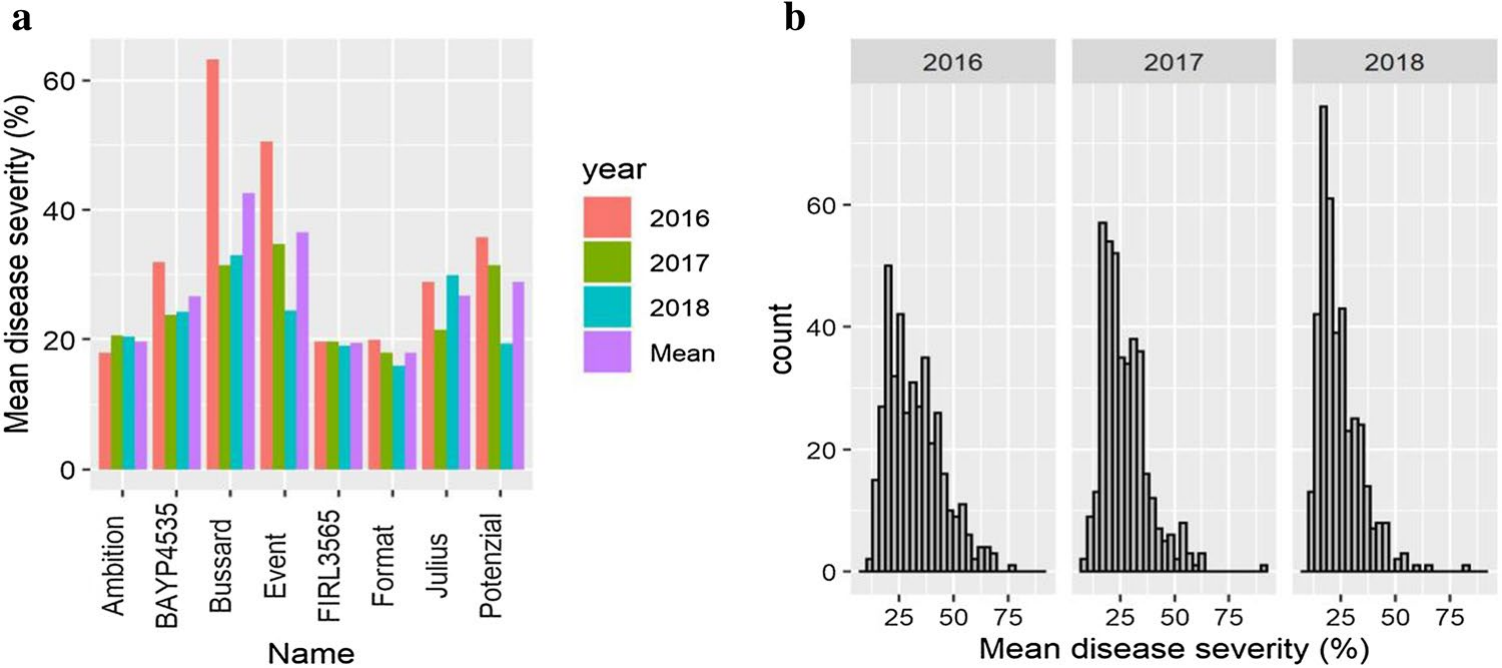
*P. nodorum* leaf blotch phenotypes for BMWpop trials at Ås, Norway, during seasons 2016, 2017 and 2018. **a** Mean leaf blotch disease severity of BMWpop founders, **b** Histograms of disease severity of BMWpop RILs in different years

The three varieties used as SNB controls performed as expected, with Tarso, Arina and Jenga showing high, medium and low SNB infection in all trials, respectively (Table S1). Among the eight BMWpop founders, Bussard and Event showed the highest mean leaf blotch disease severity (Fig. 1a). Broad and transgressive variation for leaf blotch severity was observed among BMWpop RILs (Fig. 1b). PH was not significantly correlated with leaf blotch severity in any of the three years studied, whereas DH was significantly correlated with disease severity in both 2017 (*r* = −0.31, *p* < 0.0001) and 2018 (*r* = −0.24, *p* < 0.0001) (Table 1). The mean leaf blotch severities were all significantly (*p* < 0.0001) correlated between years, with phenotypic correlation coefficients ranging from 0.36 (2017–2018) to 0.47 (2016–2017) (Table 1). As neither PH nor DH was significantly correlated with disease severity in 2016, the mean disease severities from 2016 were used directly for both QTL and haplotype analysis, while disease severity data from both 2017 and 2018 were corrected for DH effects. The mean disease severity data were not normally distributed (*p* < 0.0001), being skewed toward lower disease severity in all three years (Fig. 1b).

**Table 1:** Pearson correlation coefficients for leaf blotch disease severity, days to heading (DH) and plant height (PH) in the 2016, 2017 and 2018 season trials

	PH	DH	2016 Leaf blotch	2017 Leaf blotch
2016 leaf blotch	− 0.08	− 0.05		
2017 leaf blotch	− 0.09	− 0.31***	0.47***	
2018 leaf blotch	− 0.07	− 0.24***	0.37***	0.36***

***p < 0.0001

### Phenotypic evaluation of seedling infiltration

**Fig. 2:**
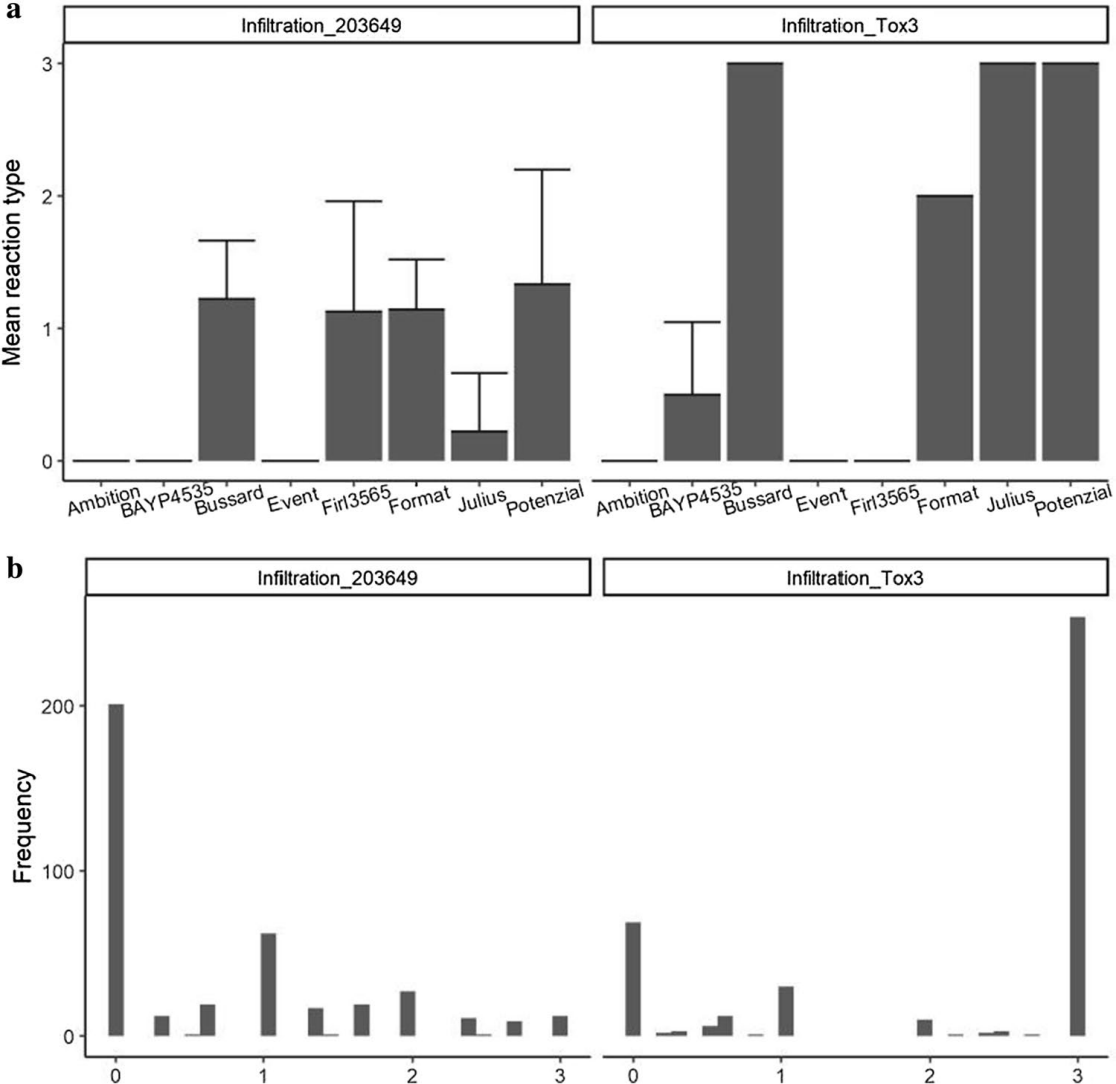
**a** Reactions of BMWpop founders to infiltration with culture filtrates of *P. nodorum* isolate 203649 (left) and Tox3 effector (right). Error bars indicate the standard deviation of mean reaction type for each parent. 9 replicates were used for infiltration with isolate 203649, while 6 replicates were used for infiltration with Tox3. **b** Histogram of the reactions of BMWpop RILs to infiltration with culture filtrates of *P. nodorum* isolate 203649 (left) and Tox3 effector (right)

Founder reactions to infiltration with culture filtrate of *P. nodorum* isolate 203649 are shown in Fig. 2a. Ambition, BAYP4535 and Event were completely insensitive (score 0). Two of the nine replicates tested for Julius showed a weak sensitive reaction (score 1), the remaining seven were completely insensitive (score 0), whereas Bussard, Firl3565, Format and Potenzial showed higher sensitivity (scores between 1 and 2). However, transgressive segregation was observed in the population (Fig. 2b): 8.4% of the RILs showed a culture filtrate sensitivity score > 2, which exceeded the sensitivity range of all eight founders (Fig. 2a). 59.4% of the RILs were insensitive (score < 1), while 32% of RILs showed intermediate reactions (1 < score < 2) (Fig. 2b).

Infiltration with the *P. nodorum* effector Tox3 identified three founders (Bussard, Julius and Potenzial) to be highly sensitive (score 3), whereas Ambition, Event and Firl3565 were completely insensitive (score 0) (Fig. 2a). Two founders showed intermediate reactions, with Format being relatively sensitive (score 2) and BAYP4535 being relatively insensitive with a mean score < 1 (Fig. 2a). Sixty-four percent of the RILs showed strong sensitivity to Tox3 (score 3), whereas 18% of the RILs showed complete insensitivity (Fig. 2b). The remaining 18% of the RILs showed intermediate sensitivity (Fig. 2b).

### Genetic analysis of SNB resistance/sensitivity

**Fig. 3:**
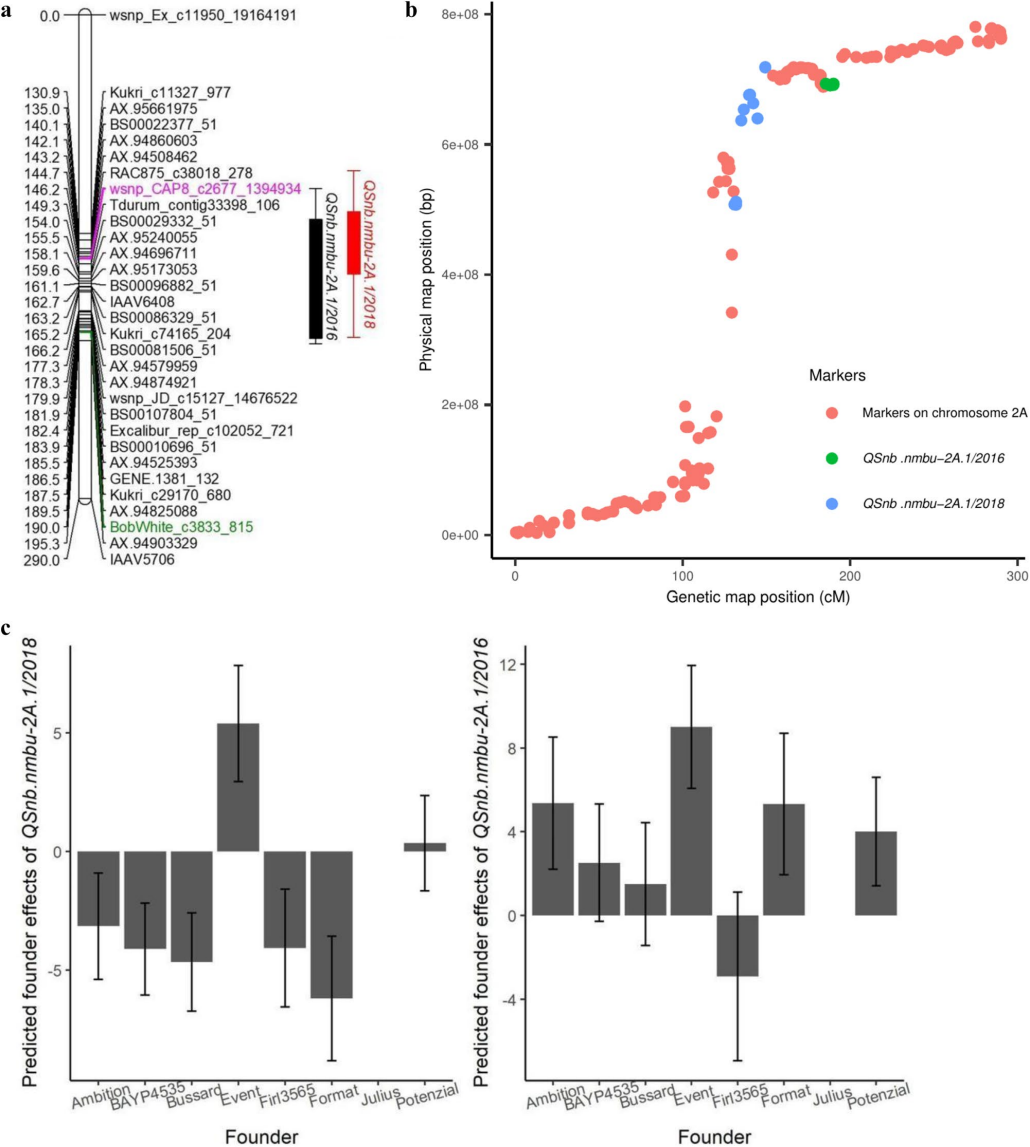
Summary information for leaf blotch QTL identified for leaf blotch on chromosome 2A in the BMWpop population. **a** Results of QTL scan using interval mapping (IM). Peak marker QTL detected in 2018 is indicated in pink, while QTL detected in 2016 are indicated in green. **b** Plot of the genetic (Stadlmeier et al. 2018) versus physical map (IWGSC RefSeq v1.0) position of SNPs mapped to chromosome 2A in the BMWpop. Markers within the support interval of *QSnb.nmbu-2A.1/2016* and *QSnb.nmbu-2A.1/2018* are indicated in green and blue, respectively. **c** Predicted founder effects for *QSnb.nmbu-2A.1/2016* and *QSnb.nmbu-2A.1/2018*, relative to the founder Julius. Error bars indicate the standard error of the estimated founder effects

Four significant QTL were detected for field leaf blotch resistance/sensitivity on chromosomes 2A, 2B, 2D and 5A, each explaining between 5 and 7% of the phenotypic variation (Table 2). In this study, two QTL were initially detected for leaf blotch resistance/susceptibility on chromosome 2A: *QSnb.nmbu-2A.1/2018* and *QSnb.nmbu-2A.1/2016* (Fig. 3a). *QSnb.nmbu-2A.1/2018* was the most significant of the two (−log_10_(*p*) = 3.4) and explained 5.4% of the phenotypic variation (peak marker *wsnp_CAP8_c2677_1394934*, located at 146.2 cM on the genetic map and 603.5 Mb on the physical map) (Table 2 and Fig. 3a, b). At this QTL, the Event allele increased the corrected disease severity relative to Julius by 5.4%, whereas alleles from all but one of the remaining founders decreased the corrected disease severity by > 3% relative to Julius—notably Format and Bussard, with disease reductions of 6.2% and 4.7%, respectively (Fig. 3c). *QSnb.nmbu-2A.1/2016* (–log_10_(*p*) = 2.8) was detected in the 2016 trial on chromosome 2A at 190 cM (692 Mb) and explained 5.2% of the phenotypic variation (Table 2 and Fig. 3a), with the allele from Event also contributing the most to susceptibility (increasing the mean disease severity relative to Julius by 9.01%). However, in contrast to *QSnb.nmbu-2A.1/2018*, the Format allele had a relatively high increasing effect on mean disease severity (Fig. 3c). *QSnb.nmbu-2A.1/2018* and *QSnb.nmbu-2A.1/2016* were firstly considered as two distinct QTL since their respective QTL peaks were located approximately 40 cM apart on BMWpop genetic map. However, on the wheat physical map, the *QSnb.nmbu-2A.1/2016* interval was located within that of *QSnb.nmbu-2A.1/2018* (Fig. 3b). Additionally, the predicted founder effect of these two QTL was similar, where Event contributed the most to disease susceptibility (Fig. 3c). As these QTL are close to the highly non-recombining region (Table 3, Fig. 3b), the balance of evidence is not sufficient to state that these two QTL are different. We therefore subsequently treat them as a single QTL here. However, further validation is required to confirm this assumption.

**Table 2:** *P. nodorum* resistance/sensitivity QTL identified in the BMWpop MAGIC population from field trials at the adult plant stage, as well as from culture filtrate infiltration and Tox3 infiltration at the seedling stage

QTL	Reference	Trait	Year	Chr	Interval (cM)	Flanking markers	Peak Marker	−log_10_(*p*)	IWGSC RefSeq v1.0 start (bp)	IWGSC RefSeq v1.0 end (bp)	R^2^ (%)	Methods used to detect QTL
*QSnb.nmbu-2A.1*		Leaf blotch	2018	2A	130.88–149.29	Kukri_c11327_977 and Tdurum_contig33398_106	wsnp_CAP8_c2677_1394934	3.4	507,691,472	718,885,511	5.4	IM, CIM (cov5, cov10), IBD
*QSnb.nmbu-2A.1*		Leaf blotch	2016	2A	185.46–190.00	AX-94525393 and BobWhite_c3833_815	BobWhite_c3833_815	2.8	688,619,335	693,294,681	5.2	IM, CIM (cov5), IBD
*QSnb.nmbu-2B.1*		Leaf blotch	2017	2B	191.41–207.19	Excalibur_rep_c66577_159 and AX-94522698	Ra_c71978_532	3.5	572,591,268	648,083,739	6.9	IM, IBD
*QSnb.nmbu-2D.1*	Phan et al. (2016), Lin et al. (2020a)	Leaf blotch	2016	2D	11.22–14.26	D_F1BEJMU01A0OMY_356 and BS00067698_51	BS00071755_51	2.6	14,636,197	15,115,231	7.0	IM, CIM (cov5, cov10), IBD
*QInf.nmbu-5A.1*		Infil_203649		5A	44.98–59.90	BS00040623_51 and BobWhite_rep_c64913_315	BS00040623_51	3.4	391,548,987	413,418,597	6.2	IM, CIM (cov5, cov10), IBD
*QSnb.nmbu-5A.1*	Friesen et al. (2009), Liu et al. (2015)	Leaf blotch	2016	5A	205.90–220.61	Excalibur_c472_914 and Tdurum_contig54785_216	Excalibur_c33923_592	3.0	558,692,780	568,272,320	6.7	IM, CIM (cov5, cov10), IBD
*QSnb.nmbu-5A.1*	Friesen et al. (2009), Liu et al. (2015)	Leaf blotch	2018	5A	207.91–220.61	Tdurum_contig44343_1039 and Tdurum_contig54785_216	RAC875_c25339_200	3.1	558,692,880	571,683,217	5.0	IM, CIM (cov5, cov10)
*QTox3.nmbu-5B.1*	Friesen et al. (2009), Downie et al. (2018), Lin et al. (2020a)	Infil_Tox3		5B	0–4.55	wsnp_Ku_c5308_9450093 and wsnp_Ex_c2459_4591587	wsnp_JD_rep_c48937_33188230	Inf	12,324,613	16,421,984	36.0	IM, CIM (cov5), IBD
*QInf.nmbu-5B.1*		Infil_203649		5B	100.27–104.80	BobWhite_c7070_196 and BobWhite_rep_c50822_462	BobWhite_c7070_196	3.8	508,795,354	516,041,245	6.0	IM, CIM (cov5, cov10), IBD
*QInf.nmbu.6A.1*		Infil_203649		6A	6.72–19.59	wsnp_Ex_c21633_30782312 and RAC875_c6135_95	Kukri_c7146_870	3.0	796,322	2,402,510	6.9	IM, CIM (cov5, cov10), IBD
*QTox3.nmbu.6A. 1*		Infil_Tox3		6A	171.15–174.69	wsnp_RFL_Contig1871_1020122 and IAAV151	wsnp_Ku_c3450_6387847	4.0	545,832,350	574,479,996	5.5	IM, IBD
*QInf.nmbu.7B.1*	Lin et al. (2020a)	Infil_203649		7B	169.77–179.91	GENE.4442_121 and BS00057323_51	wsnp_Ex_c56425_58548596	14.9	679,800,093	700,551,772	17.1	IM, CIM (cov5, cov10), IBD

Reference indicates previously published QTL predicted to co-locate with the BMWpop QTL identified. Chromosome (Chr.), proportion of the variance explained by QTL (R^2^). The –log_10_(*p*) value for *Snn3-B1* is recorded as ‘Inf’, as the p-value was 0, resulting in an error when converted to the log_10_ scale. *IM*  interval mapping, *CIM* composite interval mapping, *IBD *identity by descent. Cov5 = 5 covariates. Cov10 = 10 covariates

**Table 3:** Overview of published QTL identified for SNB in the *QSnb.nmbu-2A.1* region on chromosome 2A, based on positions on the wheat reference genome assembly (RefSeq v1.0; International Wheat Genome Sequencing Consortium (IWGSC) et al. 2018)

Marker	Population	Genetic map position (cM) ^†^	Physical map position start (bp)^‡^	Physical map position end (bp) ^‡^	*P* value	Source	QTL name
Kukri_c11327_977	BMWpop	130.88	507,691,472	507,691,373	1.42E-03	This study/2018_left	*QSnb.nmbu.2A.1*
BS00055514_51	NIAB Elite MAGIC		543,625,444	543,625,544		2014NLB-peak/(Lin et al. 2020a)	*QSnb.niab-2A.3*
**wsnp_CAP8_c2677_1394934**	BMWpop	**146.20**	**603,524,403**	**603,524,602**	**1.60E-04**	This study/**2018_peak**	*QSnb.nmbu-2A.1*
Ku_c5710_312	NIAB Elite MAGIC		605,800,158	605,800,259		2016NLB-peak/(Lin et al. 2020a)	*QSnb.niab-2A.3*
BS00062679_51	NIAB Elite MAGIC		615,287,656	615,287,757		2016NGB-peak/ (Lin. et al. 2020a)	*QSnb.niab-2A.3*
RAC875_c9372_94	NIAB Elite MAGIC		635,606,922	635,606,992		2017ULB/2018NLB-peak/ (Lin et al. 2020a)	*QSnb.niab-2A.3*
**AX-95661975**	BMWpop	**134.98**	**639,695,197**	**639,695,127**	**3.59E-04**	This study	*QSnb.nmbu-2A.1*
**RAC875_c38018_278**	BMWpop	**144.67**	**639,988,422**	**639,988,522**	**3.47E-04**	This study	*QSnb.nmbu-2A.1*
**AX-94508462**	BMWpop	**143.15**	**652,336,037**	**652,335,967**	**4.27E-04**	This study	*QSnb.nmbu-2A.1*
**BS00090569_51**	BMWpop	**136.50**	**653,680,962**	**653,680,862**	**2.43E-04**	This study	*QSnb.nmbu-2A.1*
BS00010696_51	BMWpop	183.94	688,619,335	688,619,436	7.99E-04	This study/2016_left	*QSnb.nmbu-2A.1*
BobWhite_c3833_815	BMWpop	190.00	692,850,215	692,850,316	1.80E-04	This study/2016 right_Peak	*QSnb.nmbu-2A.1*
gwm312	Calingiri × Wyalkatchem		709,048,504	709,048,682		Phan et al. 2016	*QSnb.cur-2AS1*
Tdurum_contig33398_106	BMWpop	149.29	718,885,411	718,885,511	7.52E-04	This study/2018_right	*QSnb.nmbu-2A.1*

Markers used for constructing *QSnb.nmbu-2A.1/2018* haplotypes are shown bold, while markers used for constructing *QSnb.niab-2A.3* haplotypes are underlined. *N* Norway, *U* UK, *LB* leaf blotch, *GB* glume blotch, left: left flanking marker of the QTL, right: right flanking marker of QTL, peak: peak marker of QTL. ^†^Stadlmeier et al. (2018). ^‡^International Wheat Genome Sequencing Consortium (IWGSC) et al. (2018)

**Fig. 4:**
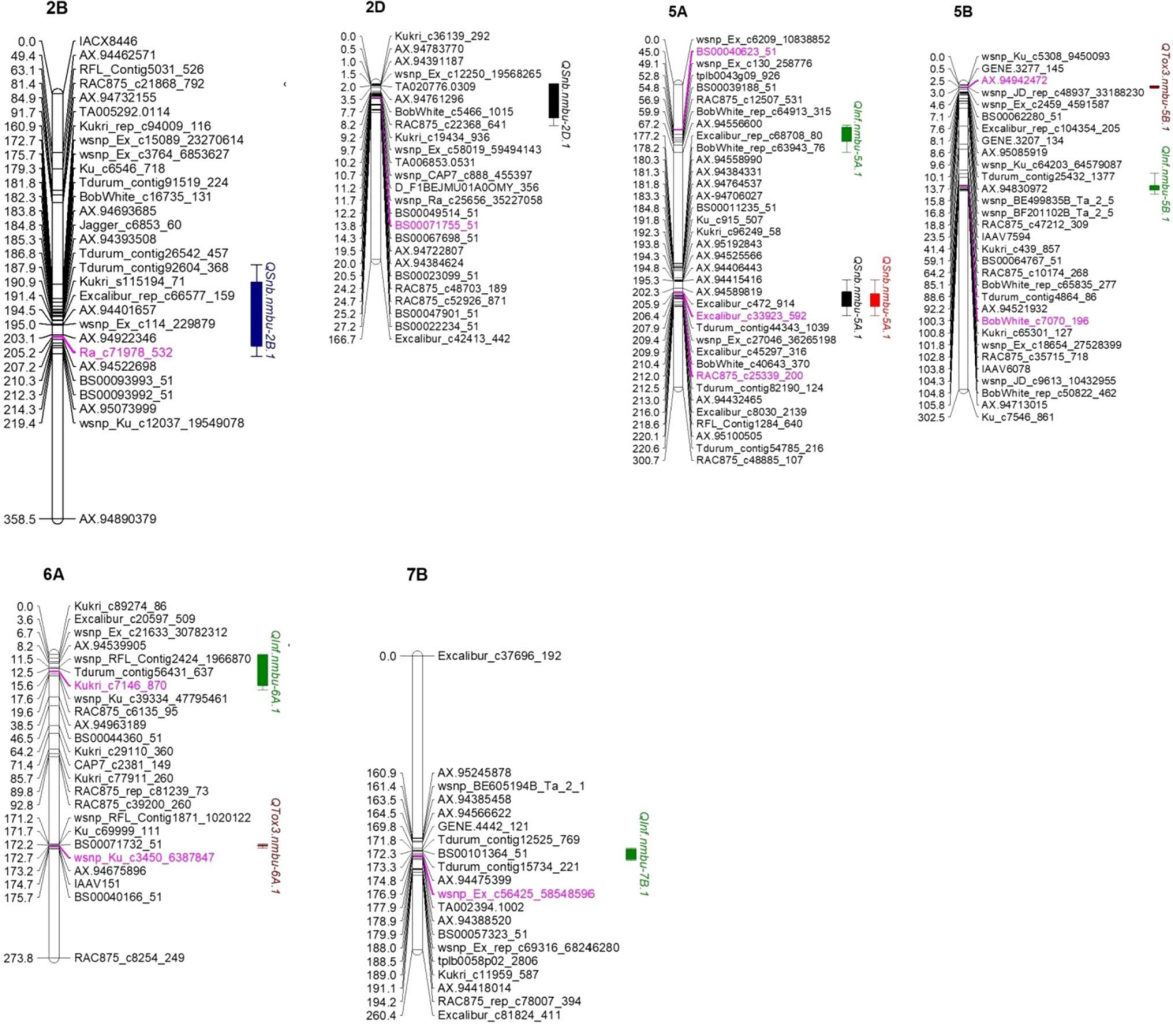
SNB QTL identified on chromosomes 2B, 2D, 5A, 5B, 6A and 7B. Results of IM are shown. QTL and permutated thresholds (−log_10_(*p*)) are colored by trait; field season 2016: black, field season 2017: blue, field season 2018: red, Culture filtrate infiltration of isolate 203649: green, Infiltration of Tox3: brown. Peak markers are indicated in pink

Another robust SNB resistance/sensitivity QTL identified in the BMWpop, *QSnb.nmbu-5A.1* on the long arm of chromosome 5A, was detected in both 2016 and 2018 and explained 6.7% and 5.0% of the phenotypic variation, respectively (Table 2, Fig. 4). The founder effects of *QSnb.nmbu-5A.1* were not conclusive for the 2 years (data not shown). *QSnb.nmbu-2B.1* (Fig. 4) on the long arm of chromosome 2B (−log_10_(*p*) = 3.5, *R*^2^ = 6.9%) and *QSnb.nmbu-2D.1* (Fig. 4) on the short arm of chromosome 2D (−log_10_(*p*) = 2.6, *R*^2^ = 7.0%) were also significant, but only detected in single years (Table 2).

### Genetic analysis of seedling infiltration

Six significant QTL were detected for greenhouse infiltration experiments, on chromosomes 5A, 5B, 6A and 7B. Two QTL were identified for Tox3 infiltration and four via infiltration using culture filtrate from *P. nodorum* isolate 203649. For Tox3 infiltration, *QTox3.nmbu-5B.1* co-located with the major *Snn3-B1* Tox3 sensitivity locus on the short arm of chromosome 5B (*p* = 0, *R*^2^ = 36%, peak marker *wsnp_JD_rep_c48937_33188230*, located at 3.05 cM/ 14.5 Mb) (Table 2, Fig. 4). In addition, another Tox3 sensitivity QTL was detected on the long arm of chromosome 6A (*QTox3.nmbu-6A.1*, −log_10_(*p*) = 4, *R*^2^ = 5.5%) (Table 2). However, *QTox3.nmbu-6A.1* was detected using IM and IBD only, and not via CIM-cov5 or -cov10. The most significant QTL for sensitivity to culture filtrate infiltration with isolate 203649 was located on chromosome 7B (*QInf.nmbu-7B.1*: −log10(*p*) = 14.9, peak marker *wsnp_Ex_c56425_58548596* at 176.9 cM/683.5 Mb) and accounted for 17.1% of the phenotypic variation (Table 2, Fig. 4). Three additional QTL less significant than *QInf.nmbu-7B.1* were also detected for sensitivity to culture filtrate infiltration on chromosomes 5A, 5B and 6A (Table 2).

### Haplotype analysis and additive effects of SNB resistance QTL *QSnb.nmbu-2A.1* and *QSnb.nmbu-5A.1*

**Fig. 5:**
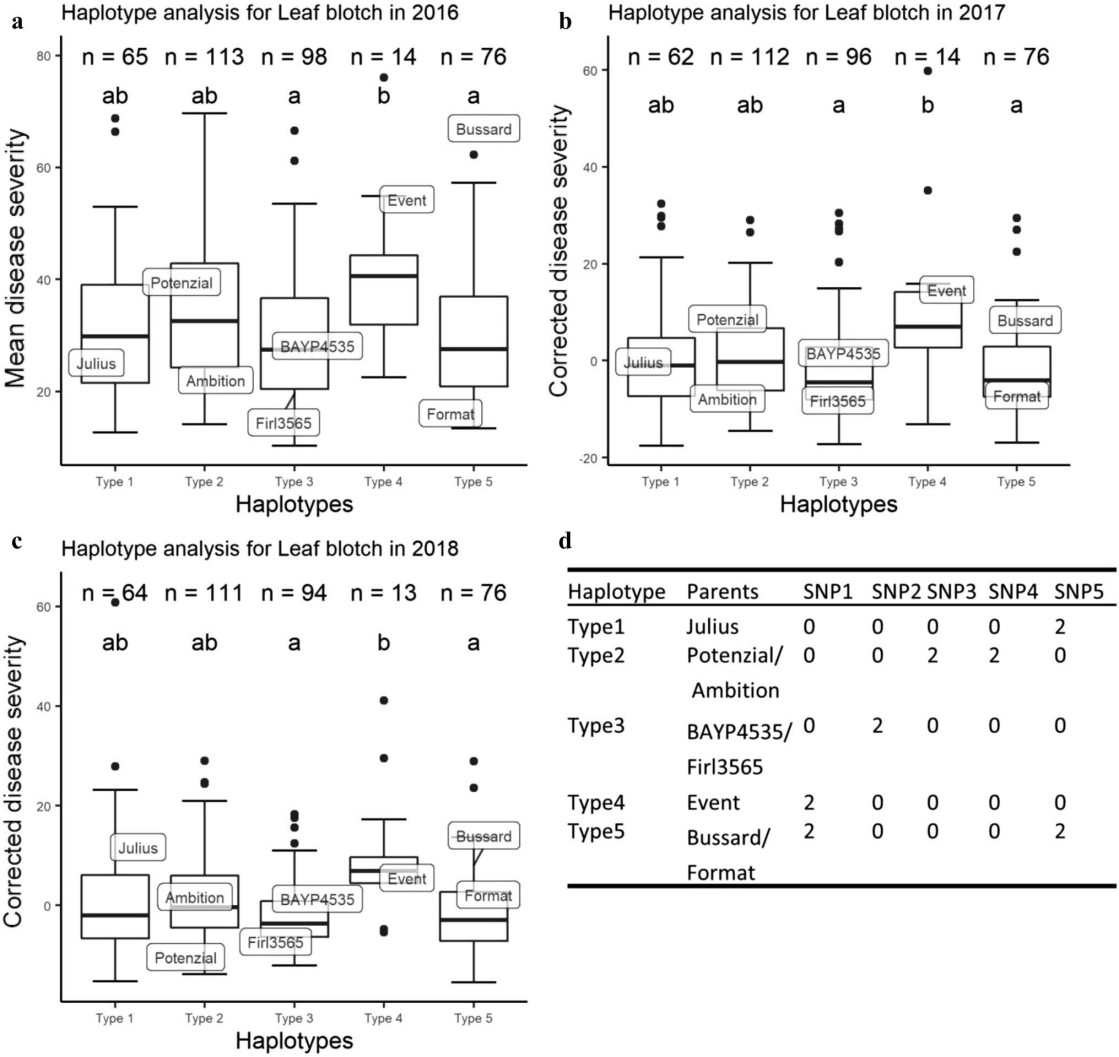
Haplotype analysis for BMWpop leaf blotch QTL *QSnb.nmbu-2A.1/2018*. **a** Haplotype analysis of mean disease severity in field season 2016. **b**, **c** Haplotype analysis of corrected disease severity in field season 2017 and 2018, respectively, and the mean disease ratings for the 8 founders are indicated. Haplotypes labeled with same letter represented no significant differences between haplotype disease severities as detected by Kruskalmc test (*p* < 0.05). **d** Genotype of each haplotype based on five SNP markers. SNP marker names are listed in order as below: *wsnp_CAP8_c2677_1394934*, *AX-95661975*, *RAC875_c38018_278*, *AX-94508462*, *BS00090569_51*

**Fig. 6:**
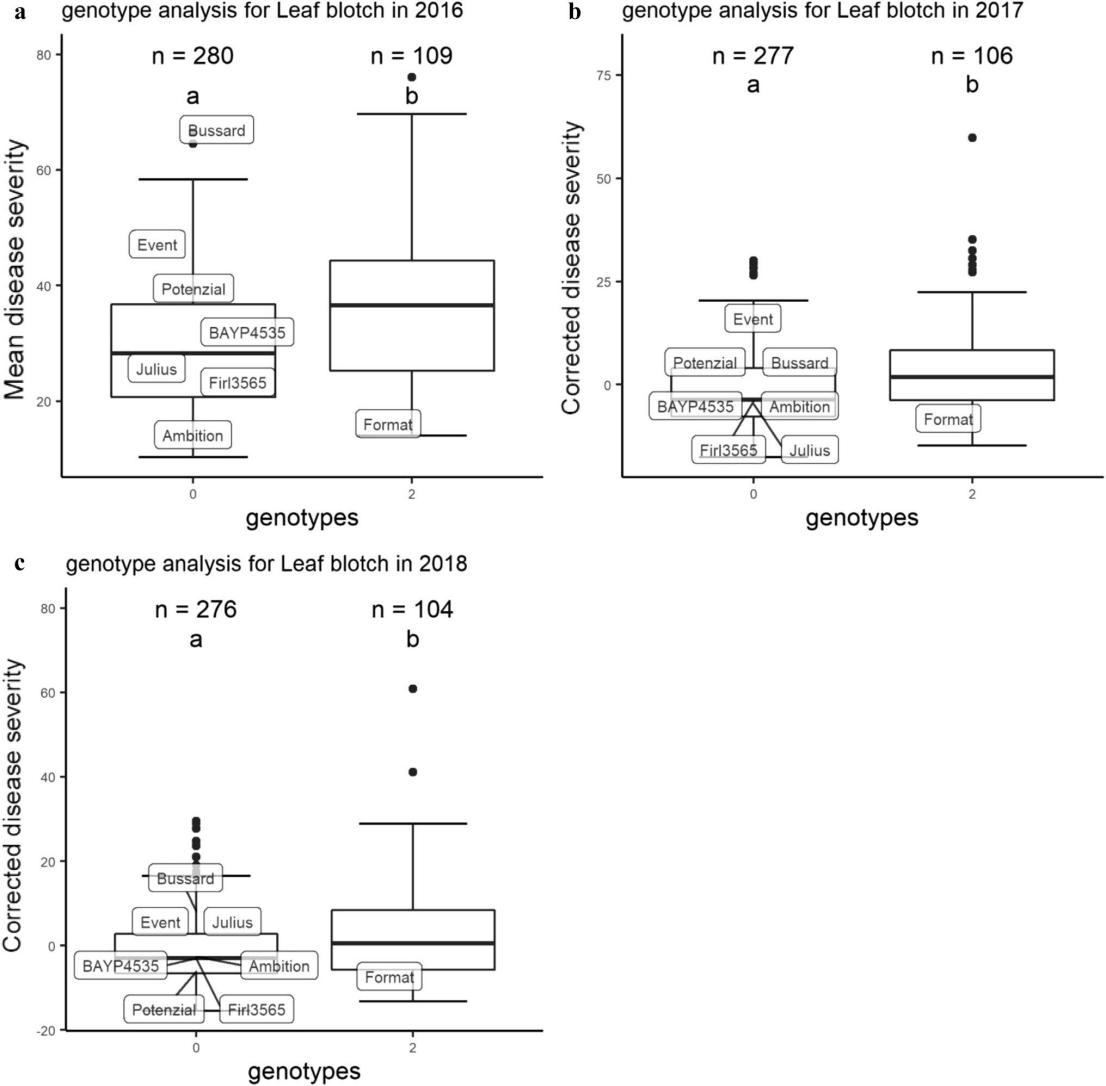
Allele effect analysis of marker *wsnp_Ex_c898_1738424* for BMWpop SNB QTL *QSnb.nmbu-5A.1*. **a** Allele effect of mean disease severity in field season 2016. **b**, **c** Allele effect of corrected disease severity in field season 2017 and 2018, respectively, and the mean disease ratings for the eight founders are indicated. Genotypes labeled with same letter represented no significant differences between haplotype disease severities as detected by Kruskalmc test (*p* < 0.05)

**Fig. 7:**
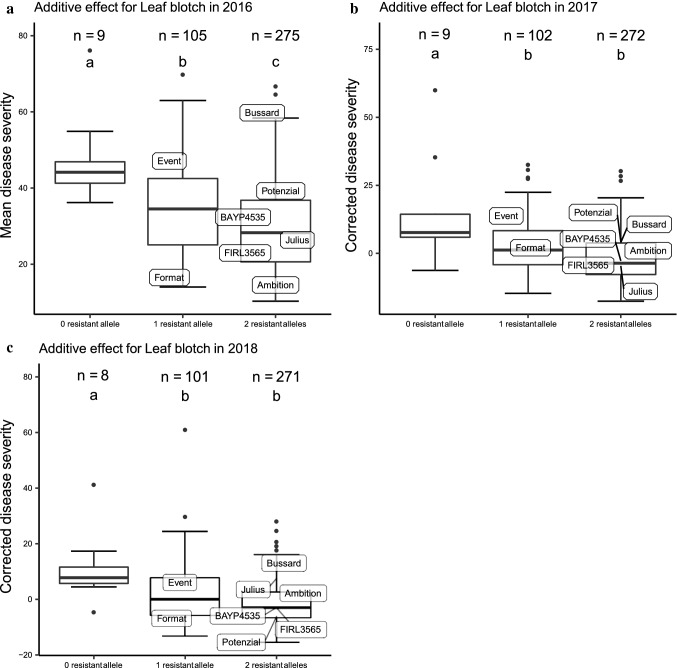
Analysis of additive effects for QTL *QSnb.nmbu-2A.1/2018* and *QSnb.nmbu-5A.1*. **a** Additive effect analysis of mean disease severity in field season 2016. **b**, **c** Additive effect analysis of corrected disease severity in field seasons 2017 and 2018, respectively. Genotypes labeled with same letter represented no significant differences between haplotype disease severities as detected by Kruskalmc test (*p* < 0.05)

Markers used for haplotype construction at *QSnb.nmbu-2A.1/2018* are listed in Table 3. In total, the five SNPs used defined five haplotypes. Consistent significant difference (*p* < 0.05) of mean/corrected disease severity was observed between not only haplotypes 3 and 4, but also between haplotypes 4 and 5 in all tested years (Fig. 5). Haplotype 4 was also always the most susceptible haplotype, with approximately 11% higher disease severity compared to haplotype 3 and haplotype 5 (Table S2). Haplotype 4 originated from the founder Event. While relatively resistant haplotype 3 originated from BAYP4535 and Firl3565, and haplotype 5 originated from Bussard and Format. The same haplotype analysis was undertaken using the phenotypic data from culture filtrate infiltration with isolate 203649, however, no significant difference of disease severity between the *QSnb.nmbu-2A.1/2018* haplotypes was observed (data not shown). Additional haplotype analysis of *QSnb.nmbu-2A.1/2016* using two significant markers defined three haplotypes, where the haplotype two originating from the founder Event always showed higher susceptibility. Significant differences between haplotype 1 and 2 (*p* < 0.05) were observed in two out of the three years tested (Fig. S1). For QTL *QSnb.nmbu-5A.1,* the allele effect of marker *wsnp_Ex_c898_1738424* (210.95 cM, 574 Mb) on SNB disease severity was significant (*p* < 0.05) in all tested years (Fig. 6). The susceptible allele was inherited from Format, while the remaining founders carry the resistance allele (Fig. 6). Figure 7 shows the decrease in SNB disease severities by stacking resistant alleles. In all tested years, significant differences in mean/corrected SNB disease severity were observed between genotypes carrying no resistant allele and those carrying 1 or 2 resistant alleles (Fig. 7). In addition, significant additive effect of stacking resistant alleles was observed in 2016 (Fig. 7a).

## Discussion

In this study, SNB field trials using a German MAGIC population (‘BMWpop’) were carried out over three seasons from 2016 to 2018 at Vollebekk field station in Ås, Norway, side by side with the trials previously reported for the UK MAGIC population (‘NIAB Elite MAGIC’) (Lin et al. 2020a). The two MAGIC populations were subjected to the same *P. nodorum* field population and similar environmental influences. However, QTL identified in the ‘NIAB Elite MAGIC’ population may not necessarily be detected in the BMWpop, despite the similar field environments the trials were conducted under. Except for cv. Ambition which originated from a Danish breeding program (Nordic Seed), all BMWpop founders were commercially released or bred for release in Germany (Stadlmeier et al. 2018), while founders of the ‘NIAB Elite MAGIC’ were all released in the UK (Mackay et al. 2014). In addition, the two MAGIC populations were genotyped using different SNP arrays, with 1335 SNP markers in common for direct comparison of genetic maps. Thus, in addition to the use of different founders between the two populations, differences in marker density, RIL numbers and crossing designs may also result in differences in power and precision with which to detect QTL. Despite all these factors, two adult plant SNB resistance/sensitivity QTL were detected in common between the two populations: *QSnb.nmbu-2A.1* and *QSnb.nmbu-2D.1*.

The BMWpop 2A QTL *QSnb.nmbu-2A.1* was identified as a robust QTL for SNB leaf blotch susceptibility in the UK MAGIC population across multiple years (*QSnb.niab-2A.3*) (Lin et al. 2020a). The *QSnb.nmbu-2A.1/2018* interval overlapped with that of *QSnb.niab-2A.3*, and the peak marker of *QSnb.nmbu-2A.1/2018* was just ~ 2 Mb away from that of *QSnb.niab-2A.3* for 2016 in Norway (Table 3). Haplotype analysis of *QSnb.niab-2A.3* has previously confirmed the robustness of this QTL across years and locations in the ‘NIAB Elite MAGIC’ population (Lin et al. 2020a). Interestingly, haplotype analysis also confirmed the consistent effect of BMWpop QTL *QSnb.nmbu-2A.1/2018* for leaf blotch susceptibility in all three tested years and *QSnb.nmbu-2A.1/2016* for two years (Fig. 5, Fig. S1). When comparing genetic maps of the two MAGIC populations, two common markers *BS00090569_51* and *RAC875_c38018_278* were found within the supporting intervals of these chromosome 2A QTL in both the UK MAGIC (*QSnb.niab-2A.3*) and German MAGIC (*QSnb.nmbu-2A.1/2018*) populations. Both markers were among the most significant markers detected for *QSnb.nmbu-2A.1/2018* and were used for constructing *QSnb.nmbu-2A.1/2018* haplotypes. *QSnb.nmbu-2A.1/2018* haplotype 4 and *QSnb.nmbu-2A.1/2016* haplotype 2, which were inherited from the founder Event, showed significantly higher disease severity (Fig. 5, Fig. S1). This observation also fitted the predicted founder effects at this QTL, with Event contributing the most to leaf blotch susceptibility (Fig. 3c). Therefore, we hypothesize that the susceptible haplotype from founders Xi19 and Rialto in the UK MAGIC population and the susceptible haplotype from Event in the German MAGIC population may carry the same susceptibility allele. However, while pedigree analysis shows that Xi19 was a result of a cross between the varieties Rialto and Cadenza, no pedigree information for Event could be identified to confirm possible common allelic origin within established wheat pedigree resources (e.g., Fradgley et al. 2019). Moreover, one flanking marker for the *P. nodorum* seedling resistance/sensitivity QTL *QSnb.cur-2AS.1* reported by Phan et al. (2016) also aligned to the interval defined by *QSnb.nmbu-2A.1* (Table 3). However, since *QSnb.cur-2AS.1* encompasses a large physical interval (from 112 to 709 Mb), further meaningful comparison is not possible.

*QSnb.nmbu-5A.1* located between 558.693–571.683 Mb on chromosome 5A was the second BMWpop QTL to be significant in more than one year. In addition, the allelic effect using marker *wsnp_Ex_c898_1738424* was significant for all tested years in our study, where only the founder Format carries the susceptibility allele (Fig. 6). Given the discriminatory nature of this SNP in our eight founders, these results highlight the potential of this marker for application in marker-assisted selection. Two adult plant SNB resistance/susceptibility QTL have previously been reported on chromosome 5A (Friesen et al. 2009; Francki et al. 2020) (Table S3). Of these, our SNB QTL *QSnb.nmbu-5A.1* (558.7–571.7 Mb) overlaps with the location of a locus conferring resistance/susceptibility to SNB in the bi-parental population BR34 × Grandin (~ 558.340 Mb) (Friesen et al. 2009). The BMWpop QTL *QSnb.nmbu-2D.1* (14.6–15.1 Mb) detected in 2016 co-located with the ‘NIAB Elite MAGIC’ QTL *QSnb.niab-2D.1* (14.8–27.8 Mb), reported by Lin et al. (2020a) to be located near the well characterized Tox2 sensitivity locus *Snn2* (6.2–12.4 Mb) (Zhang et al. 2009) and *QSnb.cur-2DS* (14.3–37.0 Mb) (Phan et al. 2016) (Table S4).

QTL *QSnb.niab-2A.3* was recently identified by culture filtrate infiltration with isolate 203649 in the ‘NIAB Elite MAGIC’ population, with the same haplotype effect observed for both field resistance and sensitivity to infiltration and inoculation with isolate 203649 (Lin et al. 2020a). This is the same isolate as used here for CF infiltration of the BMWpop. However, no CF QTL on chromosome 2A were identified in the BMWpop. Rather, CF infiltration identified QTL on chromosomes 5A, 5B and 7B. The reason why CF with isolate 203649 did not identify the 2A QTL in the BMWpop even though an SNB leaf blotch QTL was detected at this locus is not clear, and could be due to various reasons such as interactions between the CF effector complement and the genetic background of the BMWpop, or differences in the expression of effectors within different batches of CF. Interestingly, the ʻweakʼ ‘NIAB Elite MAGIC’ SNB resistance QTL *QSnb.niab-7B.2* (−log_10_(*p*) = 2.91, *R*^2^ = 5.83%) was detected as a major BMWpop QTL for culture filtrate infiltration (*QInf.nmbu-7B.1*: −log_10_(*p*) = 14.9, *R*^2^ = 17.1%). The QTL interval of *QInf.nmbu-7B.1* is located within that of *QSnb.niab-7B.2* on the physical map, and their peak markers were located ~ 4 Mb apart (Table S5). Phan et al. (2016) found that Tox3 expression levels were increased when the Tox1 gene was knocked out in *P. nodorum* isolate SN15, indicating that Tox3 expression was suppressed by Tox1. It is possible that the expression of the uncharacterized *P. nodorum* effector which interacted with the *QInf.nmbu-7B.1* locus in the BMWpop may have suppressed the expression of the uncharacterized effector which has been previously shown via culture filtrate infiltration in the UK MAGIC population to interact with *QSnb.niab-2A.3* (Lin et al. 2020a). Recently, Peters Haugrud et al. (2019) also reported that effects caused by the NE-host inverse gene for gene interactions varied from epistatic to additive and depended on the genetic backgrounds of both host and pathogen. Therefore, the different genetic background of the host populations might result in the phenomenon where *QSnb.niab-2A.3* was detected in ‘NIAB Elite MAGIC’ via culture filtrate infiltration but not in the BMWpop, despite infiltration with culture filtrate using the same isolate. In addition, the expression level of the uncharacterized NE which interacted with *QSnb.niab-2A.3* in ‘NIAB Elite MAGIC’ might be low, and thus the detection of this interaction could be masked by the interaction of *QInf.nmbu-7B.1* and the other uncharacterized NE. Similar to CF infiltration results, even though BMWpop segregates for the *Snn3-B1* locus and the Tox3 gene is common in the Norwegian *P. nodorum* population (Ruud et al. 2018), the *Snn3-B1* QTL was not detected in our field testing. This observation could be explained by a hypothesis proposed by Peters Haugrud et al. (2019) where *P. nodorum* isolates might not express all of the NE genes they harbor. Alternatively, the pathogen population occurring in the field lacked Tox3 or an epistatic effect may exist between unknown NE-*Snn* and Tox3-*Snn3-B1* interactions under the field condition. Clearly the situation is relatively complex, and further studies are required to disentangle *P. nodorum* effector–wheat susceptibility interactions as well as *P. nodorum* effector–effector interactions in order to determine which SNB QTL are dependent on host-NE interactions.

In order to identify additional host genetic loci controlling sensitivity to new *P. nodorum* effectors, and to compare the locations of these to SNB QTL, we screened the BMWpop for sensitivity to CF from a local Norwegian *P. nodorum* isolate that lacked the three cloned effectors (ToxA, Tox1 and Tox3). None of the CF sensitivity QTL co-located with SNB leaf blotch QTL in this population. This could be because there were no effectors present in the CF, or because the BMWpop founders do not contrast for sensitivity to the hypothesized effectors. Alternatively, the isolate we used may not be representative of those responsible for the natural field infection conditions under which we investigated SNB resistance. Indeed, as our recent research finds the Norwegian *P. nodorum* population to be genetically diverse (lacking in obvious genetic populations substructure, and collectively containing all eight possible combinations for the presence/absence of the three cloned effector genes) (Lin et al. 2020b), it is likely that this latter hypothesis is correct. Future work investigating possible correlation between NE-*Snn* interactions and SNB in the BMWpop would therefore benefit from CF assessment using isolates obtained from the naturally infected experimental trials under study. In addition to screening for CF sensitivity, we also phenotyped the BMWpop for sensitivity to Tox3. This identified the major sensitivity locus *Snn3-B1*, as well as a minor sensitivity QTL on chromosome 6A (6A: 545.832–574.480 Mb). Anchoring this minor QTL to the wheat physical map finds it to overlap with two previously identified QTL. The first is the *Snn6* locus, which confers sensitivity to Tox6 (6A:574.222–606.980 Mb) (Gao et al. 2015), while the second is a seedling resistance QTL *QSnl.ihar-6A* (6A:579.126–583.269) (Arseniuk et al. 2004). Of note, a possible relationship between genetic loci controlling sensitivity to Tox3 and other effectors can be identified with the co-localisation of the Tox3 sensitivity locus *Qsnb.fcu-4BL* (4B: 608.252–657.496 Mb) (Phan et al. 2016) with the Tox5 sensitivity locus *Snn5* (4B: 608.252–640.977 Mb) (Friesen et al. 2012). Direct links between the molecular pathways controlling different wheat sensitivity to *P. nodorum* effectors would intuitively make sense; however, whether this is true in practice remains to be further explored.

In summary, adult plant SNB resistance/susceptibility QTL on chromosomes 2A and 2D, and seedling infiltration resistance/susceptibility QTL on chromosome 7B previously identified in the UK ‘NIAB Elite MAGIC’ population were validated here in the German eight-parent BMWpop winter wheat MAGIC population. In the BMWpop, both haplotype effect at *QSnb.nmbu-2A.1* and allele effect at *QSnb.nmbu-5A.1 *were significantly associated with field SNB susceptibility and significant across years, highlighting the robustness of these QTL. In addition, significant differences in SNB disease severity detected in 2016 between genotype groups showed evidence that the effect of these two field-relevant QTL was additive. As SNB resistance in the field is a complicated quantitative trait, validating field resistance QTL using an independent mapping panel provides robust evidence of the efficacy of target QTL in diverse genetic backgrounds. This knowledge should underpin efficient selection for beneficial SNB resistance alleles across multiple loci in wheat breeding programs, and will assist further research toward the identification of the functional allele(s) underlying these genetic loci.

## Electronic supplementary material

Below is the link to the electronic supplementary material.

Supplementary file1 (DOCX 223 kb)

Supplementary file2 (XLSX 65 kb)
