# Supplementary material for: Identification and cross-validation of genetic loci conferring resistance to Septoria nodorum blotch using a German multi-founder winter wheat population
Source: Theor Appl Genet. 2020 Oct 12;134(1):125–42. doi: 10.1007/s00122-020-03686-x (PMC7813717; doi:10.1007/s00122-020-03686-x)

Table S1 Mean disease severities (%) of check varieties tested from 2016 to 2018.

| Name | Control Type | 2016 | 2017 | 2018 |
| --- | --- | --- | --- | --- |
| Jenga | Resistant | 16 | 15 | 15 |
| Arina | Moderately resistant | 41 | 34 | 31 |
| Tarso | Susceptible | 48 | 51 | 37 |

Table S2 Mean of corrected disease severity of each haplotype in different field environments (seasons 2016, 2017 and 2018).

| Haplotype | Inherited from BMWpop founder | Mean disease severity % (2016) | Corrected disease severity % (2017) | Corrected disease severity % (2018) |
| --- | --- | --- | --- | --- |
| 1 | Julius | 31 | 1 | 1 |
| 2 | Ambition, Potenzial | 35 | 1 | 1 |
| 3 | BAYP4535, Firl3565 | 30 | -2 | -2 |
| 4 | Event | 41 | 10 | 9 |
| 5 | Bussard, Format | 30 | -2 | -2 |

Table S3 Comparison of marker physical positions for BMWpop and previously published QTL on chromosome 5A. Peak markers are indicated in bold. left: left flanking marker of the QTL, right: right flanking marker of QTL, peak: peak marker of QTL. ^†^Stadlmeier et al. (2018). ^‡^IWGSC (2018).

| Marker ID | Population | Genetic map position^†^ | Physical map position start (bp) ^‡^ | Physical map position end (bp) ^‡^ | Reference | Source | |
| --- | --- | --- | --- | --- | --- | --- | --- |
| **BS00040623_51** | BMWpop | 44.98 | 391548987 | 391548887 | This study/ *QInf.nmbu-5A.1* | Infil_203649_left/peak | |
| BobWhite_rep_c64913_315 | BMWpop |  | 413418697 | 413418597 | This study/ *QInf.nmbu-5A.1* | Infil_203649_right | |
| **BobWhite_rep_c63943_76** | BMWpop |  | 547415426 | 547415526 | This study/ *Qsnb.nmbu-5A.1* | 2016_peak/right | |
| AX-94706027 | BMWpop | 183.29 | 548234891 | 548234821 | This study/ *Qsnb.nmbu-5A.1* | 2016_right | |
| barc151 | BR34×Grandin |  | 558340037 | 558340252 | (Friesen et al., 2009)/ *QSnb.fcu-5AL* | Adult/ seedling leaf |  |
| Tdurum_contig54785_216 | BMWpop | 220.61 | 558692780 | 558692880 | This study/ *Qsnb.nmbu-5A.1* | 2016_right/2018_right |  |
| IWB67424 | 120 hard red winter wheat (HRWW) cultivars |  | 565753008 | 565752908 | (Liu et al., 2015) | Seedling leaf |  |
| **Excalibur_c33923_592** | BMWpop |  | 568156970 | 568157070 | This study/ *Qsnb.nmbu-5A.1* | 2016_peak |  |
| Excalibur_c472_914 | BMWpop | 205.9 | 568272220 | 568272320 | This study/ *Qsnb.nmbu-5A.1* | 2016_left |  |
| **RAC875_c25339_200** | BMWpop |  | 571683315 | 571683217 | This study/ *Qsnb.nmbu-5A.1* | 2018_peak |  |
| Tdurum_contig44343_1039 | BMWpop | 207.91 | 573589801 | 573589901 | This study/ *Qsnb.nmbu-5A.1* | 2018_left |  |
| IWA675 | GWAS 232 lines (global origin) |  | 609872751 | 609872951 | (Francki et al., 2020) | Adult leaf |  |

Table S4 Comparison of physical marker positions for BMWpop QTL *QSnb.nmbu-2D.1* on chromosome 2D. Peak markers are indicated in bold. left: left flanking marker of the QTL, right: right flanking marker of QTL, peak: peak marker of QTL. ^†^IWGSC (2018).

| Marker | Population | Physical position start (bp) ^†^ | Physical position end (bp) ^†^ | Source |
| --- | --- | --- | --- | --- |
| Xcfd56 | BR34×Grandin | 6158983 | 6158963 | (Zhang et al., 2009) /*Snn2* |
| Xcfd51 | BR34×Grandin | 12360665 | 12360684 | (Zhang et al., 2009) /*Snn2* |
| BobWhite_c5466_1015 | BMWpop | 14261151 | 14261251 | 2016_left |
| cfd36 | Calingiri × Wyalkatchem | 14362782 | 14362981 | (Phan et al., 2016)/ *QSnb.cur-2DS* |
| BS00029208_51 | NIAB Elite MAGIC | 14897896 | 14897996 | (Lin et al., 2020a) peak |
| BS00071755_51 | BMWpop | 15115131 | 15115231 | 2016_peak |
| BS00047901_51 | BMWpop | 15967348 | 15967448 | 2016_right |
| wsnp_JD_rep_c63957_40798083 | NIAB Elite MAGIC | 20768482 | 20768682 | (Lin et al., 2020a) |
| BobWhite_c59161_181 | NIAB Elite MAGIC | 27859904 | 27859806 | (Lin et al., 2020a) |
| wPt-669517 | Calingiri × Wyalkatchem | 37053347 | 37053740 | (Phan et al., 2016)/ *QSnb.cur-2DS* |

Table S5 Comparison of physical marker positions for BMWpop QTL *QSInf.nmbu-7B.1* and *QSnb.niab-7B.2* on chromosome 7B*.* Peak markers are indicated in bold. ^†^IWGSC (2018).

| Marker | Population | Physical map position start (bp) ^†^ | Physical map position end (bp) ^†^ | QTL name |
| --- | --- | --- | --- | --- |
| Kukri_c15912_860 | NIAB Elite MAGIC | 673961429 | 673961530 | *QSnb.niab--7B.2* |
| GENE.4442_121 | BMWpop | 679800093 | 679799993 | *QInf.nmbu-7B.1* |
| **wsnp_Ex_c56425_58548596** | **BMWpop** | **683513848** | **683513648** | ***QInf.nmbu-7B.1*** |
| **BS00077956_51** | **NIAB Elite MAGIC** | **687304661** | **687304762** | ***QSnb.niab-7B.2*** |
| BS00057323_51 | BMWpop | 687591650 | 687591750 | *QInf.nmbu-7B.1* |
| Excalibur_c50612_409 | NIAB Elite MAGIC | 700551671 | 700551772 | *QSnb.niab-7B.2* |

Table S6 Permutated p=0.05 significance threshold in each environment.

| Trait | Threshold |
| --- | --- |
| Leaf blotch in 2016 | 2.6 |
| Leaf blotch in 2017 | 2.8 |
| Leaf blotch in 2018 | 2.2 |
| Infiltration with cultural filtrate of isolate 203649 | 3.0 |
| Infiltration with Tox3 effector | 3.5 |

Fig. S1 Haplotype analysis for BMWpop leaf blotch QTL *QSnb.nmbu-2A.1/2016.* (a) Haplotype effect of mean disease severity in field season 2016. (b-c) Haplotype effect of corrected disease severity in field season 2017 and 2018, respectively, and the mean disease ratings for the eight founders are indicated. Haplotypes labeled with same letter represented no significant differences between haplotype disease severities as detected by Kruskalmc test (p < 0.05).


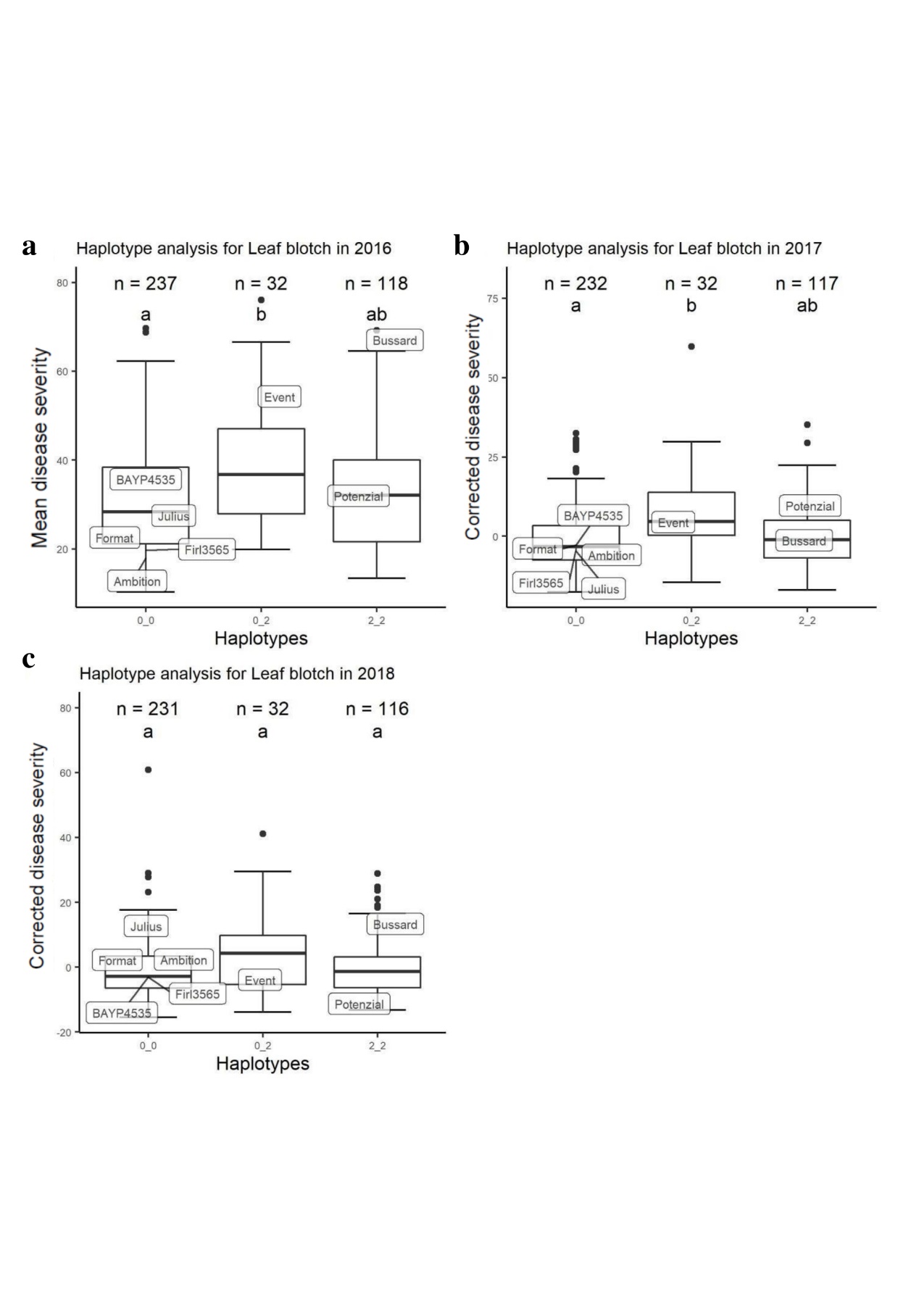

Supplement: Supplementary file 1 — Supplementary file1 (DOCX 223 kb) [file 122_2020_3686_MOESM1_ESM.docx]
